# Correction: FDPP–HA as a theranostic agent for cancer-targeted fluorescence imaging and photodynamic therapy

**DOI:** 10.1039/d3ra90050a

**Published:** 2023-06-12

**Authors:** Pingping Liang, Jinjun Shao, Qianyun Tang, Weili Si, Qiang Wang, Qi Zhang, Xiaochen Dong

**Affiliations:** a Key Laboratory of Flexible Electronics (KLOFE), Institute of Advanced Materials (IAM), Jiangsu National Synergetic Innovation Center for Advanced Materials (SICAM), Nanjing Tech University (NanjingTech) Nanjing China iamxcdong@njtech.edu.cn; b School of Pharmaceutical Sciences, Nanjing Tech University (NanjingTech) Nanjing China zhangqi@njtech.edu.cn; c College of Chemistry and Molecular Engineering, Nanjing Tech University (NanjingTech) Nanjing China

## Abstract

Correction for ‘FDPP–HA as a theranostic agent for cancer-targeted fluorescence imaging and photodynamic therapy’ by Pingping Liang *et al.*, *RSC Adv.*, 2017, **7**, 37369–37373, https://doi.org/10.1039/C7RA06551E.

In the original article in Fig. 6, incorrect H&E staining images were inserted for the control group (liver) and illumination group (spleen and kidney). The corrected versions are included below. The authors apologise for any inconvenience caused.

**Fig. 6:**
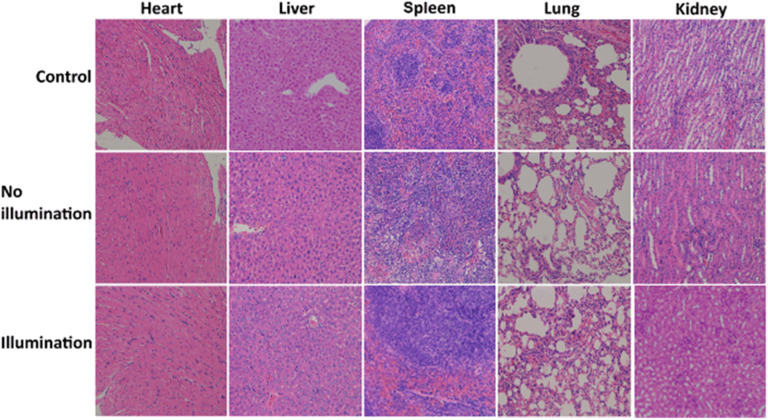
Photographs of H&E staining for major organs including heart, liver, spleen, lung, and kidney obtained from three groups after 30 days of treatment.

The results and conclusions of this paper are not affected by this correction.

The Royal Society of Chemistry apologises for these errors and any consequent inconvenience to authors and readers.

## Supplementary Material

